# Posterior lower uterine segment caesarean hysterotomy, an innovative surgical technique to avoid classical hysterotomy: Case report and discussion

**DOI:** 10.1177/2050313X241284037

**Published:** 2024-10-01

**Authors:** Kyungu M Kime, Lisa Bazzett-Matabele, Mercy N Nassali, G Justus Hofmeyr

**Affiliations:** 1 Department of Obstetrics and Gynaecology, University of Botswana, Gaborone, Botswana; 2 Effective Care Research Unit, University of the Witwatersrand and Walter Sisulu University, East London, South Africa

**Keywords:** Caesarean birth, inaccessible lower uterine segment, posterior lower uterine segment hysterotomy, intrauterine device, case report

## Abstract

A 40-year-old patient underwent laparotomy at term gestation for a 25-cm lower abdominal mass arising from the lateral wall of the uterus, with an extensive secondary blood supply from the lower uterus and bladder, preventing access to the anterior lower uterine segment. The gravid uterus was exteriorised over the patient’s thighs. A transverse posterior lower uterine segment hysterotomy was performed and a healthy 2920 g baby was delivered. A copper T 380A intrauterine device was placed at the fundus and the insertion tube passed through the cervix for retrieval after the surgery. A Foley catheter uterine tourniquet was applied to allow bloodless excision of the tumour. Histological examination confirmed a leiomyoma with extensive hyalinisation. The few prior reports of posterior lower uterine segment caesarean hysterotomy were mainly for uterine torsion or placenta accreta spectrum. It is useful to be aware of this simple though counter-intuitive approach when faced with technical difficulties.

## Introduction

The rate of caesarean births is increasing globally, as are surgical complications. A not infrequent surgical difficulty is that of restricted access to the anterior lower uterine segment, due to, for example, adhesions from previous surgery, tumours in the lower uterine segment and anterior placenta praevia with placenta accreta spectrum (PAS). The traditional solution is to perform an upper uterine segment hysterotomy.^1,2^ Disadvantages include increased short-term surgical complications and increased risk of uterine rupture in a subsequent pregnancy, which may occur even before labour. This is particularly important in low-resource settings where the patient’s access to prompt care in future pregnancies may be uncertain. Although at the time of the case reported below we were not aware that posterior lower uterine segment hysterotomy had been used previously, we subsequently identified four prior reports of posterior lower uterine segment hysterotomy performed in the presence of 180° torsion of the uterus, of which two were purposeful^3,4^ and two were inadvertent, the torsion being undiagnosed.^5,6^ Another inadvertent posterior hysterotomy was performed in a case of uterine sacculation.^7^ In one case of 180° uterine torsion, a classical hysterotomy was performed,^8^ and in another, the uterus was exteriorised to correct the torsion and do an anterior lower segment hysterotomy.^9^ In five patients with placenta accreta sequence, amniotic fluid drainage was used to facilitate exteriorisation of the gravid uterus through a transverse abdominal incision, followed by anterior or posterior hysterotomy.^10^ In all the cases of posterior lower segment hysterotomies, good outcomes were reported. On the other hand, classical (upper uterine segment) hysterotomy is associated with considerable morbidity.^11^

## Case presentation

Our patient is a 40-year-old mother of two boys ages 14 and 13 years, having had an early miscarriage in 2007, admitted at 37 weeks of gestation in her fourth pregnancy (gravida 4, term 2, preterm 0, abortion 1 and living children 2). Her mother suffered from diabetes. She was socially stable and employed in the security industry. She had been diagnosed with a pelvic tumour the previous year on ultrasound but had withdrawn from care when advised that surgery might entail a hysterectomy, as she wished to have another child. She reported no other medical symptoms.

### Clinical findings

She appeared well-looking, with no remarkable general clinical findings. Examination revealed a large cystic/solid tumour occupying the lower abdomen. The gravid uterus was displaced upwards and posteriorly to the left hypochondrium.

### Timeline

2021: Diagnosed with pelvic tumour.
21 January 2022: ‘Booked’ for current pregnancy.
12 May 2022: Admitted.
18 May 2022: Surgery.
21 May 2022: Signed consent for case report and was discharged home.
Surgery: Posterior lower uterine segment hysterotomy and excision of tumour 6 weeks post-surgery: Well at follow-up.

### Diagnostic assessment

Ultrasound examination at 22 weeks showed a complex predominantly cystic lesion in the pelvis measuring 25.6 × 14.1 × 19.7 cm. The possibility of an ovarian tumour was considered. Haematological and renal function tests were within the normal ranges.

### Therapeutic interventions

Surgery was performed at 38 weeks of gestation under spinal analgesia. A vertical abdominal incision revealed a large solid/cystic tumour occupying the lower abdominal cavity (see Supplemental Video 1). The tumour was within the right broad ligament with an 8 cm diameter attachment to the right upper segment of the uterus, and extending down to the retro-vesical space. It had a profuse secondary blood supply from the broad ligament and lower uterine segment and bladder anteriorly, and also from the omentum, which was dissected free. The gravid uterus was displaced postero-laterally and superiorly to lie in the left hypochondrium. The tumour could not be mobilised from the uterus easily due to extensive vascularity, and the lower uterine segment could not be accessed anteriorly.

**Figure 1.**
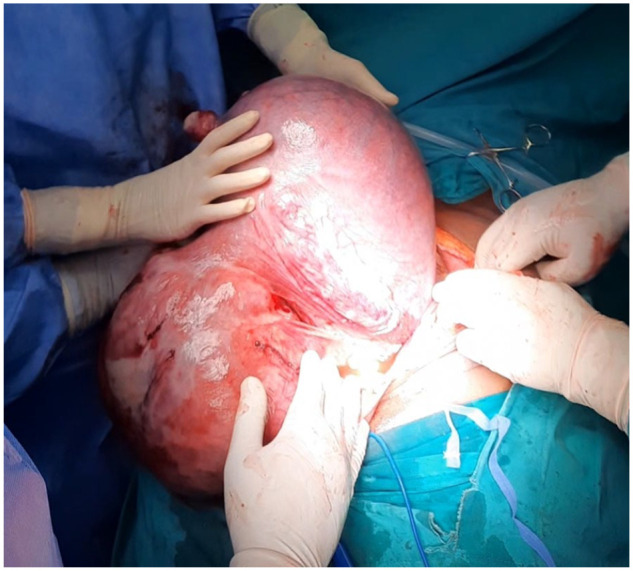
Exteriorised dextro-rotated gravid uterus, anterior view with tumour in the foreground.

**Figure 2.**
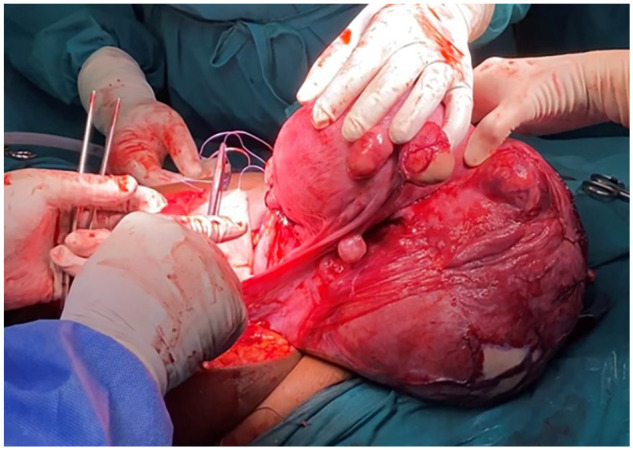
Repairing the posterior lower uterine segment hysterotomy with interrupted figure 8 sutures.

To avoid the immediate and long-term risks of classical upper uterine segment hysterotomy, the vertical abdominal incision was extended and the uterus exteriorised and inverted over the patient’s thighs (Figure 1). A transverse posterior lower uterine segment hysterotomy was performed. A healthy female infant weighing 2920 g was delivered without difficulty, with Apgar score^12^ of 9 and 10 at 1 and 5 min, respectively. The placenta was delivered by cord traction. A copper-T intrauterine device (IUD) was placed at the fundus. To protect the threads, the end of the insertion straw was directed through the cervix and retrieved vaginally after the operation. The hysterotomy was closed with interrupted figure 8 braided polyglycolic acid sutures, crossed within the uterus (Figure 2).

A Foley catheter uterine tourniquet was applied around the broad ligaments and the lower uterine segment, which provided an almost bloodless field during the separation of the tumour from the uterus and removal, which was uneventful. The tumour was sub-serosal and did not require enucleation or myometrial resection. The origin of the tumour from the right side of the uterine upper segment was sequentially clamped and the myometrial pedicles ligated. The tumour was separated from the broad ligament with diathermy and the secondary blood supply ligated.

### Follow-up and outcomes

Our patient and her baby were discharged on day 3 after surgery in good condition and with no need for a blood transfusion. At 6-week follow-up, both were well, and she expressed satisfaction with her care. Ultrasound showed a well-positioned copper-T IUD in the endometrial cavity.

The histology report was of a 27 × 20 × 20 cm leiomyoma with extensive hyalinisation.

## Discussion

Surgeons not infrequently encounter unusual anatomic barriers to routine surgical procedures and need to be prepared to problem solve on the spot with novel improvisations. In our case, although counter-intuitive, posterior lower uterine segment hysterotomy proved to be a remarkably straightforward and effective solution. We acknowledge the lack of magnetic resonance imaging as a limitation of our preoperative diagnostic work-up. The predominantly cystic appearance of the mass on ultrasound examination was atypical for uterine leiomyoma.

Avoidance of classical hysterotomy may be particularly important in low-resource settings, where the 6%–10% risk of rupture of a classical uterine scar^13^ (one-third before the onset of labour^14^), may be particularly dangerous for women with limited access to obstetric facilities.^15^ In our case, the posterior hysterotomy was very straightforward and closure was no different from an anterior lower segment incision, other than that there was no need to reflect the peritoneum and bladder.

Single-layer interrupted hysterotomy closure has been reported to reduce the risk of subsequent scar dehiscence^16^ as well as subsequent PAS,^17^ and is used routinely by several clinicians in our unit. A video demonstrating this technique for anterior lower segment closure as well as our novel technique for IUD insertion at caesarean birth is available at https://www.youtube.com/watch?v=ToWkp3z0_Vg (Supplemental Video 2).

Our case illustrates the value of a Foley catheter uterine tourniquet to reduce blood loss during uterine surgery.^18^ We have also described the use of a Foley catheter tourniquet to control placental bleeding in advanced extrauterine pregnancies.^19^ We describe a novel method we have developed to promote the correct positioning of the threads during caesarean placement of a copper-T IUD.

## Conclusion

Based on our case and the reported literature, we suggest that when the anterior lower uterine segment is inaccessible, for example, due to adhesions, vascularity, placenta praevia accrete spectrum or pelvic masses, consideration be given to exteriorised posterior lower segment hysterotomy as an alternative to classical hysterotomy. It is important for obstetricians to be aware of this simple procedure as it is not a routinely taught or intuitive approach.

## Patient perspective

On informal interaction, our patient appeared very satisfied with her care and the outcome. We did not formally record an account of the case from her perspective.
